# Polyhydroxybutyrate Production from Natural Gas in A Bubble Column Bioreactor: Simulation Using COMSOL

**DOI:** 10.3390/bioengineering6030084

**Published:** 2019-09-16

**Authors:** Mohsen Moradi, Hamid Rashedi, Soheil Rezazadeh Mofradnia, Kianoush Khosravi-Darani, Reihaneh Ashouri, Fatemeh Yazdian

**Affiliations:** 1 Department of Chemical Engineering, Faculty of Engineering, Islamic Azad University North Tehran Branch, Tehran 1651153311, Iran; khakeraheyar110@yahoo.com (M.M.); srezazadeh69@gmail.com (S.R.M.); 2 Biotechnology Group, School of Chemical Engineering, College of Engineering, University of Tehran, Tehran 11155-4563, Iran; hrashedi@ut.ac.ir; 3 Department of Food Technology Research, Faculty of Nutrition Sciences and Food Technology/National Nutrition and Food Technology Research Institute, Shahid Beheshti University of Medical Sciences, Tehran 19395–4741, Iran; 4 Department of Environment, Faculty of Environment and Energy, Science and Research Branch, Islamic Azad University, Tehran 1477893855, Iran; reihaneh.ashouri@yahoo.com; 5 Department of Life Science Engineering, Faculty of New Science & Technology, University of Tehran, Tehran 1417466191, Iran

**Keywords:** bubble column bioreactor, COMSOL, microorganism, PHB, simulation

## Abstract

In this study, the simulation of microorganism ability for the production of poly-β-hydroxybutyrate (PHB) from natural gas (as a carbon source) was carried out. Based on the Taguchi algorithm, the optimum situations for PHB production from natural gas in the columnar bubble reactor with 30 cm length and 1.5 cm diameter at a temperature of 32 °C was evaluated. So, the volume ratio of air to methane of 50:50 was calculated. The simulation was carried out by COMSOL software with two-dimensional symmetric mode. Mass transfer, momentum, density-time, and density-place were investigated. The maximum production of biomass concentration reached was 1.63 g/L, which shows a 10% difference in contrast to the number of experimental results. Furthermore, the consequence of inlet gas rate on concentration and gas hold up was investigated Andres the simulation results were confirmed to experimental results with less than 20% error.

## 1. Introduction

The facility of use and desirability of plastic materials properties led to their growing utilization in packaging and food industries [1]. At present, 30% of urban waste includes plastic waste. The extremely long durability of plastic (about three hundred years) has led to many environmental problems and destroyed the attractiveness of cities and nature. Burning polymeric lesions causes air pollution and according to the type of material used in their preparation, gases, such as hydrogen cyanide and other hazardous gases are released into the environment [1,2]. On the other hand, recycling has a lot of economic problems and costs. Since 1970, with the decline of the landfill problem worldwide, the issue of the use of biodegradable polymers was raised [3]. Polymers can be generally classified into two major biodegradable and non-degradable groups [4]. Non-degradable polymers, such as polyethylene, polypropylene, and polystyrene are produced from monomeric sources of oil and are resistant to environmental issues. Biodegradable polymers are separated according to their constituent components, their preparation process or their application. Biodegradable plastics or bioplastics with properties, such as biodegradability, environmental compatibility, the ability to produce renewable sources, less energy consumption in production, the production of water and carbon dioxide when degrading, compete with conventional plastics obtained from oil, especially with declining demand in the global market [4,5,6,7].

Amongst the biodegradable plastics, polyhydroxyalkanoates (PHAs) were considered due to the similarity of these materials with conventional plastics. The flexibility and expandable strength of PHA are similar to that of polypropylene and polystyrene polymers [2,8]. The agglomeration of PHAs in the cell can be enhanced by creating inhomogeneous growth conditions by limiting some nutrients like the source of nitrogen, phosphorus or sulfate, by reducing the concentration of oxygen, or by increasing the fraction of carbon to nitrogen in the feed [9,10].

One of the most important PHAs is polyhydroxy butyrate (PHB), which forms in the form of intracellular granules in different microorganisms. The high price of these biopolymers has led to limitations in their use of petrochemical polymers [11]. The main factor influencing the final cost of these polymers is the carbon origin, the carbon substrate yield, the method of fermentation, and its extraction method [12,13]. 

PHB can be synthesized by microorganisms with a special application in food packing [14]. However, their use is presently limited owing to the high production cost. PHB production cost is related to several key factors including the substrate, chosen strain, cultivation strategy, and downstream processing. The utilization of smart and cheap [14,15,16,17,18,19,20,21,22] modeling [23], proper bioreactors, experimental design [24,25], and the development of a new recovery method [24,25,26], as well as chances for their competition in the global market have recently been addressed. Efforts were made to optimize the growth of *Ralstonia eutropha* NRRL B14690 in the existence of nutrients, which would not only decrease the production cost of PHB but also help in increasing productivity [9]. 

Methane is the most suitable substrate for the production of PHB, both natural gas and biogas, between the most widely used carbon sources. Natural gas includes 85–90% of methane and is also produced by methane-producing bacteria in biological degradation of organic matter [27,28,29,30]. In many European countries, methane produced in low-cost biotechnology is accessible while in the United States, methane production units of urban waste are increasing rapidly; therefore, switching from PHB to natural gas to replace biodegradable plastics is necessary [7,31,32].

One way to enhance the production of PHB from natural gas is to use new bacterial types in a hydrodynamic biosystem. In a study, the PHB from natural gas was studied. After selecting the appropriate method, the effect of two key parameters of methane to air ratio and nitrogen on the production of PHB in a bubble pillar reactor revealed that both factors had a significant effect on the PHB density. The production of PHB by *Methylocystis* (*M.*) *hirsuta* was obtained in contrast to other methanotrophic bacteria to increase metabolism. After sampling from southern oilfields, suitable microorganisms were isolated for the production of PHB from natural gas (as a carbon source). Then, according to the Taguchi model, optimal conditions for the production of PHB from natural gas were appraised in the bubble column reactor [33]. The results showed the growth of microorganisms and the production of PHB in the existence of methane in the liquid phase. The variables affecting the production of PHB include temperature, air volume to methane gas, nitrogen source pH, phosphate source, age of inoculation, and culture medium [7,34]. 

In this study, first, the studies conducted in line with PHB production are conducted for familiarity and knowledge of the operation of the bioreactor activity. Then, the process is simulated using COMSOL software to find optimal circumstances and to reach a precise view for the production of PHB in a bubbler bioreactor. After this stage, the process of production of PHB in the bioreactor is investigated. Since no study was published in, the field of simulating the performance of the Methylocystis bacteria in the production of PHB in a bubble column bioreactor, the function of microorganisms in the production of PHB is simulated, which precipitates the production process and reduces costs using the results of the experiment.

## 2. Materials and Methods 

In order to examine the simulation of the process by using COMSOL software (Version 5.2: downloady.ir) and the production review in the bioreactor, first, a number of effective simplifying assumptions were selected:

- The temperature of the system is always constant at 32 °C.
- The physical properties of the solute with time are discarded.
- The velocity of all compounds in the same phase is equal.
- The culture medium and the microorganism mixture are considered as a single phase.

### 2.1. Reaction Kinetics

The production of PHB involves a type of methanotrophic bacterium, such as *M. hirsuta*, which is an aerobic and gram-negative bacteria that can produce PHB from the serum pathway. This methylotrophic bacterium of type II is used to investigate the production of PHB in the bioreactor, and the mathematical model equations provided by Equation (1) [35]:

(1) $$r_{\mathrm{PHB}}=\frac{{\mathrm{dC}}_{\mathrm{PHB}}}{\mathrm{dt}}={\;\mu C}_{\mathrm{PHB}}-K_{d}C_{\mathrm{PHB}}$$

while $C_{\mathrm{PHB}}$ is the dry weight of cell (g/L), *K_d_* is the cell death rate (s/1), and μ is specific growth rates for cell mass (1/s) (Appendix A). The modular kinetic model for producing PHB by Equation (2):

(2) $$\mu =\mu_{max}\left(\frac{s}{K_{s}+s}\right),$$

In Equation (2), μ*_max_* is the maximum specific growth rate of microorganisms (1/s), S is the concentration of limited substrate for growth (g/L), and K_S_ is the Monod constant (g/L). The kinetic of the Monod causes the substrate outlet concentration to be low in the CSTR because the K_S_ is usually small. In order to create a delay phase, it is suggested that *K_d_* changes with time in Equation (3):

(3) $$K_{d}=K_{d}\left(\infty \right)\left(1-\exp\left(\alpha t\right)\right)$$

α is the reversal of fixed cell death time (s^−1^), *K_d_* (∞) is the infinite cell death rate (s^−1^), and *t* is the time in seconds. It should be noted that for the implementation of the simulation reaction, the following simple reaction to the metabolic reactions is used for simulation:

6(CH_4_) +NH_3_+7(O_2_) → C_5_H_7_NO_2_+CO_2_ +10 (H_2_O)

### 2.2. The Equation of the Governing Model

The bubble flow model with two phases (diffused air in the liquid phase) was used in this study. In this model, the equations of continuity, momentum, and energy are solved for each step. The equation of motion is calculated by Equation (4) [34,35]:

(4) ϕlρl∂ul∂t+ϕlρl(ul·∇)ul=∇·[−pl+ϕlμl(∇ul+(∇ul)T−23(∇·ul)l)]+ϕlρlg+F

In this equation, $u_{l}$ indicates the velocity value in (m/s), p is the pressure in (Pa), and *ϕ* is the volume fraction indicated with m^3^/m^3^. ρ is density value with kg/m^3^, g is the gravity unit with m/s^2^, F is (N/m^3^), μl is the dynamic velocity of the liquid replaced Pa.s in the equation. The values I and g, respectively, show the values of the liquid phase and the gas phase. The right-hand side of Equation (4) shows all forces that involve gradient, pressure, stress, adhesion, gravity, and force between the two phases, such as pulling, lifting, and virtual collective forces. In this study, a tension force is involved in the model. Based on the explanation, the continuity equation is written as Equation (5):

(5) $$\frac{\partial }{\partial t}\left(\phi_{l}\rho_{l}+\phi_{g}\rho_{g}\right)+\nabla .\left(\phi_{l}\rho_{l}\mu_{l}+\phi_{g}\rho_{g}\mu_{g}\right)$$

and the gas phase transfer equation is calculated as in Equation (6):

(6) $$\frac{\partial \phi_{g}\rho_{g}}{\partial t}++\nabla .\left(\phi_{g}\rho_{g}\mu_{g}\right)=-m_{\mathrm{gl}}$$

m_gl_ is the mass transfer rate from gas to liquid (kg/m^3^). The gas velocity ug is equal to the sum of the velocity of Equation (7):

(7) $$U_{g}=U_{l}+U_{\mathrm{slip}}+U_{\mathrm{drift}}$$

U_slip_ is the relative velocity among phases and U_drift_ is the drift velocity. The physics relation calculates the density of gas from the ideal gas law by Equation (8):

(8) $$\rho_{g}=\frac{\left(p+p_{\mathrm{ref}}\right)M}{\mathrm{RT}}$$

M is the molecular weight of the gas (kg/mol), R is the ideal gas constant (J/(mol·K) 3/3141472), and T is the temperature (K). p_ref_ is a scalar variable being 1 at (1 at or 101.325 Pa) as a default. While a drift velocity is calculated by Equation (9)

(9) $$U_{\mathrm{drif}}=\frac{\mu \nabla \phi_{g}}{\rho \phi_{g}}$$

μ is the effective viscosity, which causes it to fall. By putting Equations (9) and (7) in (6), we will have Equation (10):

(10) $$\frac{\partial \phi_{g}\rho_{g}}{\partial t}=\nabla \cdot \left(\phi_{g}\rho_{g}\left(U_{l}+U_{\mathrm{slip}}\right)\right)=\nabla \cdot \left(\frac{\mu \nabla \phi_{g}}{\rho \phi_{g}}\right)-m_{\mathrm{gl}}$$

The drift velocity of the transfer equation is introduced in the gas transfer equation. This means that the gas transport equation is actually implemented in the physics interface. The equation of the bubble flow equation is relatively simple but it can indicate non-physical behavior. An artificial accumulation of bubbles, for example, is at the base of the walls in which the pressure gradients raise the bubbles while the bubbles have no place to go and there is no model for modifying the amount of gas fraction to grow. In order to prevent this, μ is set to μl for laminar. The only clear effect in most cases when the bubble flow equations are applicable is that the non-physical accumulation of bubbles reduces. The small effective viscosity in the transfer equation for φ_g_ has beneficial effects on the numerical properties of the equation system. 

### 2.3. Simulation Operations

**Figure 1:**
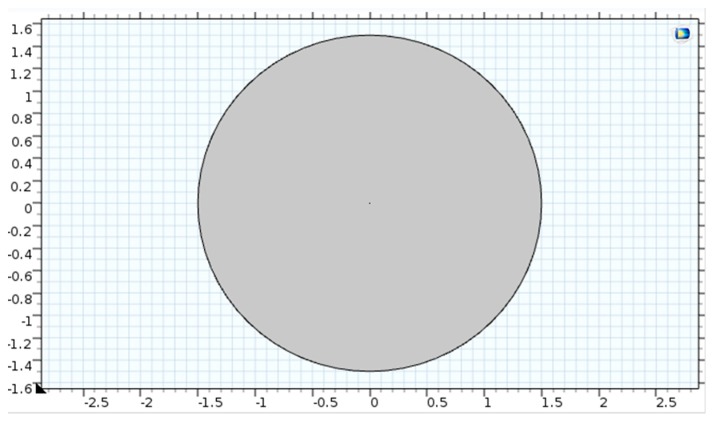
A schematic view of the system geometry.

In order to define the system, the properties and repercussions contained in COMSOL software were given to the simulation system. COMSOL simulation software has a complete set of information, properties, and constancy of materials, and if there is no specific information for a system, it can be found with the help Perry’s handbook and enter into the physics of the problem. The geometry of the system was cylindrical with a radius of 0.015 m and a stanchion at the end of the cylinder with a radius of 0.1 mm. In order to prevent computing and convergence, and in view of the symmetry in the given geometry, the problem was solved in the symmetric two-dimensional mode, which did not have an effect on the overall solution due to the application of boundary conditions. All three components of water, methane, and ammonium were regarded as a phase. In this way, the liquid environment indicates the environment of the wastewater in which the ammonium and methane are soluble, and the properties of the bacteria are applied to the environment. The reaction occurred to produce biomass of an irreversible first-degree reaction with a reaction constant $k_{1}=10^{-5}$ in form of Va → W with the reaction rate, $r=k_{1}C_{\mathrm{va}}$. The system temperature was set to 305.15 K. Since the environment is water, the incompressible flow with density and dynamic viscosity, respectively, were regarded as 1000 kg/m^3^ and 10^–3^ Pa.s. The initial pressure level (P_0_) was considered zero and the permeation coefficient of the environment 10^–9^ m^2^/s applied. The system under the boundary conditions of sleep with the equation u = 0 means that the system boundary was fixed and the simulation conducted in the symmetric two-dimensional mode. Figure 1 shows a schematic view of system geometry in the two-dimensional mode.

### 2.4. Resolution Independence Analysis of Numerical Grid

The number of model components is designed to meet the conditions of numerical resolution independence from the computational grid size and gradually increases the number of components. The resolution independence test of the computational grid to a point in which the difference in response is less than 5%. The number of components in resolution independence tests of the numerical grid is given in Table 1. 

### 2.5. Meshing 

**Figure 2:**
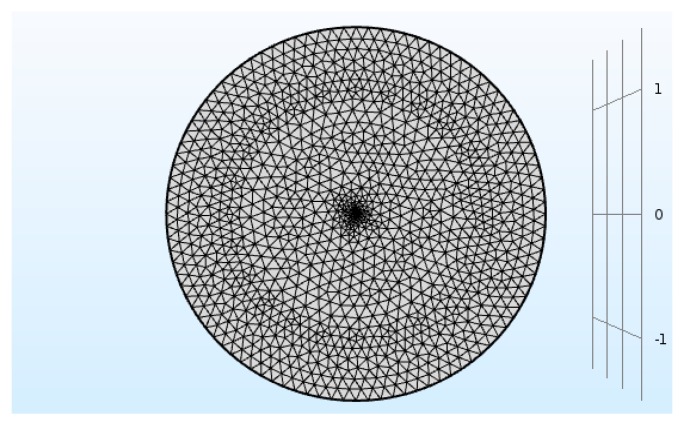
A schematic view of the geometry meshing with 34,269 components.

At this stage, the geometry of the system was considered under meshing. In a two-dimensional model, the symmetrical mesh arrangement was selected based on the free triangular model and fine mesh size. The geometry of this system was split into 34,279 components. In Figure 2, a schematic view of the system meshing is presented in a two-dimensional mode.

## 3. Results

In the production of PHB from natural gas, the objective of optimizing the composition of the culture medium is to provide important and sensitive food, increase the yield of the product, prevent product degradation, and decrease the formation of harmful side effects. 

### 3.1. The Results of Resolution Independence Analysis of Numerical Grid

As indicated in Table 2, three large, medium, and fine grid sizes were applied separately in the model and the final concentration of the biomass in each mesh size was studied to examine the sensitivity of the computational grid. Table 3 indicates the final concentration of biomass in the last step of solving equations in three computational grids. 

### 3.2. Concentration of Biomass

#### 3.2.1. Concentration Contour

**Figure 3:**
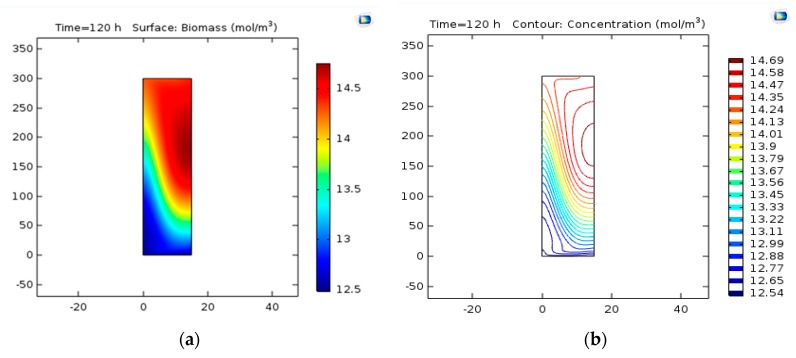

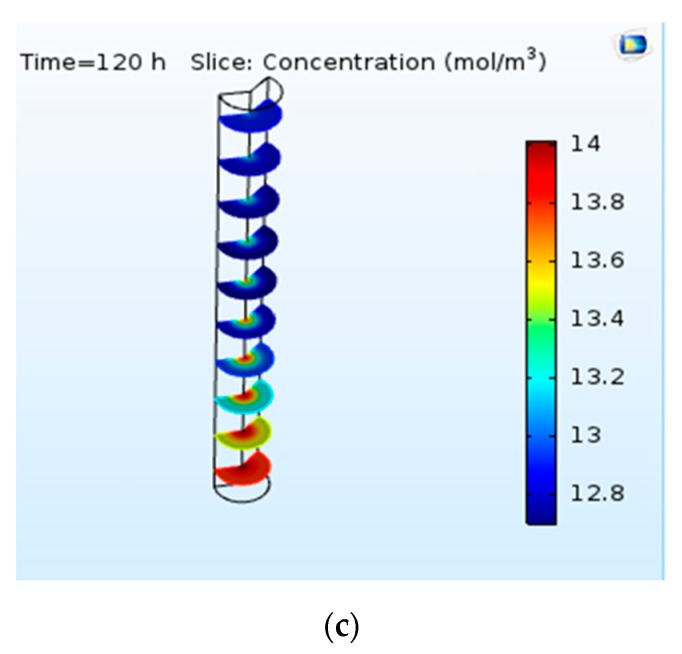
(**a**) The concentration surface contour of biomass. (**b**) The concentration contour of biomass. (**c**) The slice concentration contour of biomass.

**Figure 4:**
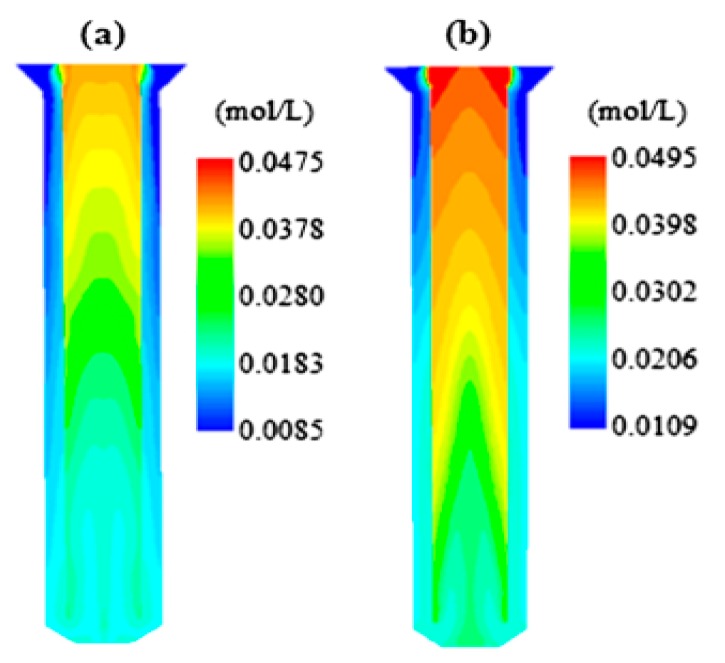
The concentration contour of polyhydroxy butyrate (PHB) at time (**a**) *t* = 2min and (**b**) *t* = 10 min [36].

Biomass (C_5_H_7_NO_2_) is generated under the applied conditions according to the reaction from Va → W. After the simulation, the concentration contour was determined at 14 min. Figure 3 displays the concentration contour of biomass. Figure 3 shows the concentration gradient is from the center toward the wall. As indicated in Figure 4, the concentration contour at time *t* = 2 min and *t* =10 min can be seen for the circulation and mixing of PHB [36].

#### 3.2.2. Concentration Variations versus Time

**Figure 5:**
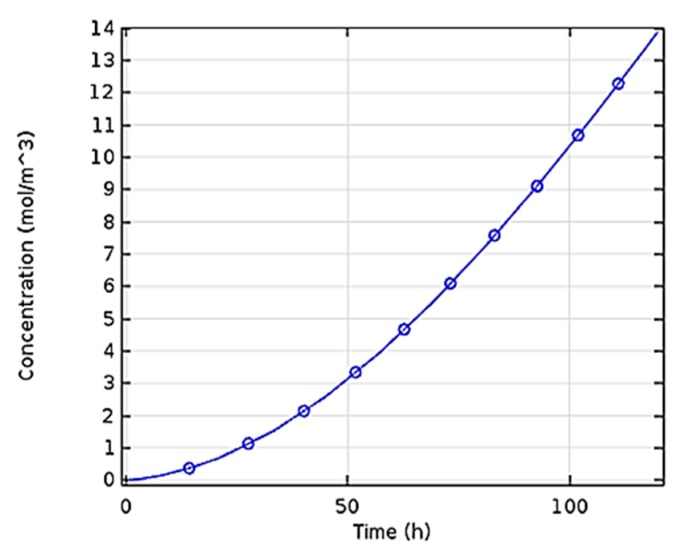
Changes in the concentration of biomass with time.

**Figure 6:**
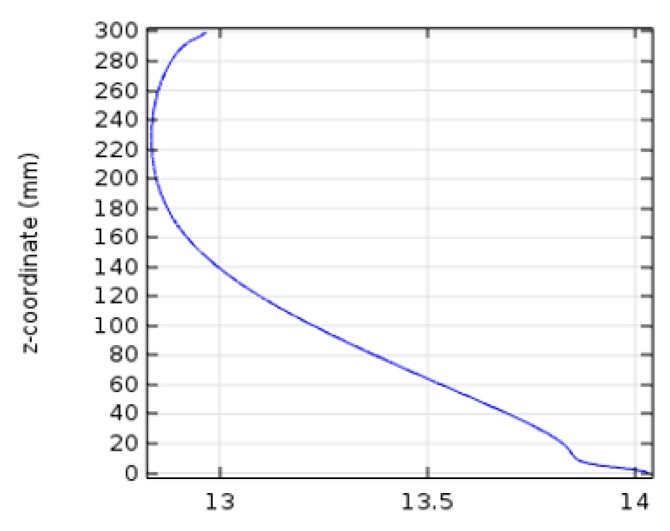
Linear changes of the reactor location concentration.

The changes in the concentration of biomass were studied with time and the results are shown in Figure 5. As can be seen in this curve, the final concentration of biomass is almost 14 mol/m^3^, which is less than 5% in comparison to laboratory values (14.5-mol/m^3^). Figure 6 indicates concentration-location changes in the two-dimensional model. As can be observed, the concentration at the beginning of the reactor is maximized because of the rapid reaction.

### 3.3. Velocity Contour

**Figure 7:**
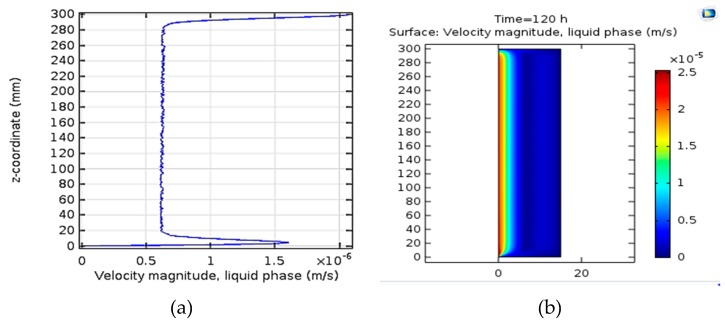
(**a**) Spatial changes of velocity in the liquid phase along the reactor. (**b**) Surface spatial changes of velocity in the liquid phase along the reactor.

Figure 7a,b indicate the results of spatial changes of fluid velocity in the bioreactor. On the right side of Figure 7, the velocity assigned to each color is represented.

### 3.4. Analysis of Variations in the Input Gas Velocity

**Figure 8:**
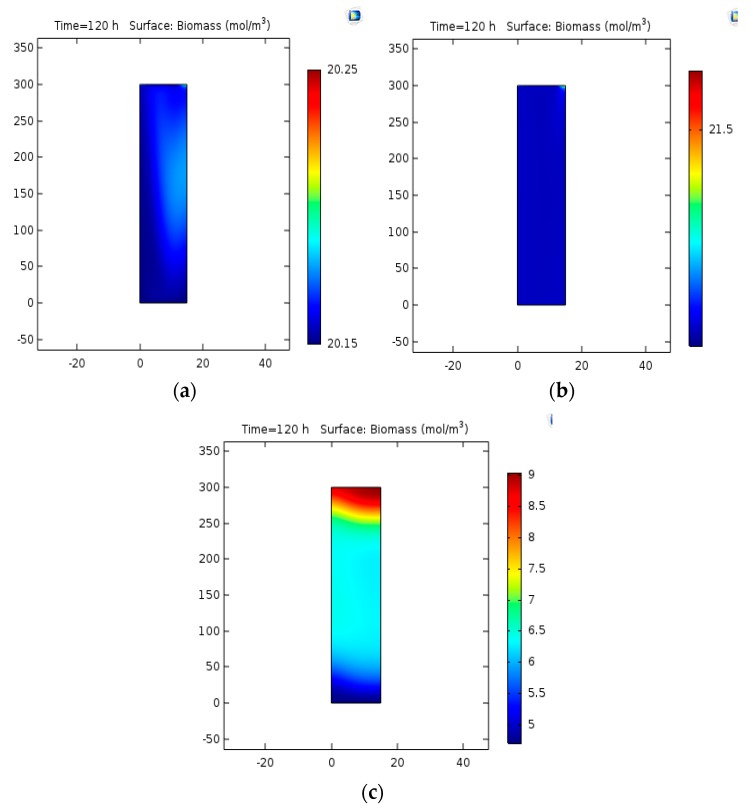
(**a**) Biomass concentration at the gas velocity of 0.0015 m/s, (**b**) biomass concentration at the gas velocity of 0.065 m/s, (**c**) biomass concentration at the gas velocity of 0.15 m/s.

The gas flow rate inside the reactor will influence the number of bubbles and the mass transfer rate. Thus, the simulation was studied at four different velocities of 0.0015, 0.065, and 0.15 m/s. The results are displayed in Figure 8a–c. 

### 3.5. Effect of Changing the Bubble Diameter on the Concentration

**Figure 9:**
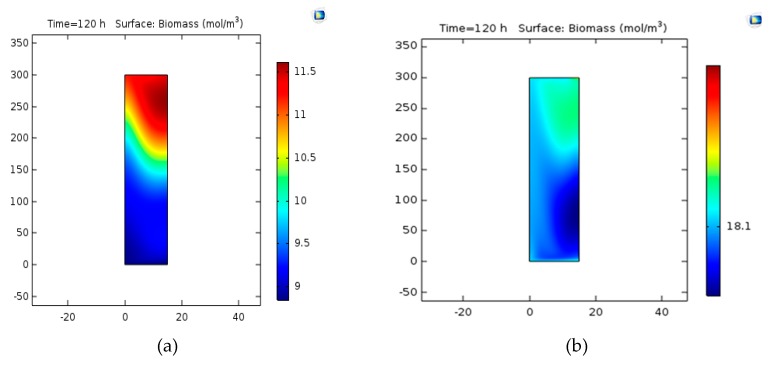
(**a**) Biomass concentration with a bubble diameter of 3.5 mm. (**b**) Biomass concentration with a bubble diameter of 1.5 mm.

In Figure 9a,b, the change of concentration of the biomass can be observed with a bubble diameter of 3.5 and 1.5 mm. 

### 3.6. Pressure Analysis

**Figure 10:**
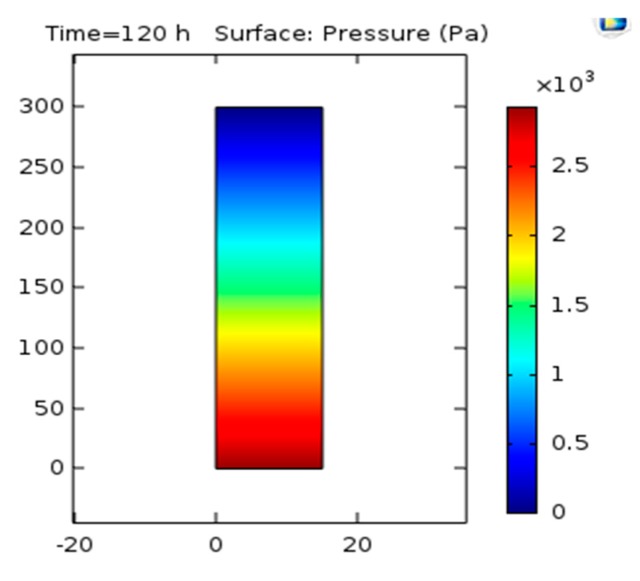
Pressure contour in two-dimensional mode.

The relation between the amount of gravity and the bubbles are directed which are effected on pressure changes as presented in Figure 10.

### 3.7. Gas Accumulation

**Figure 11:**
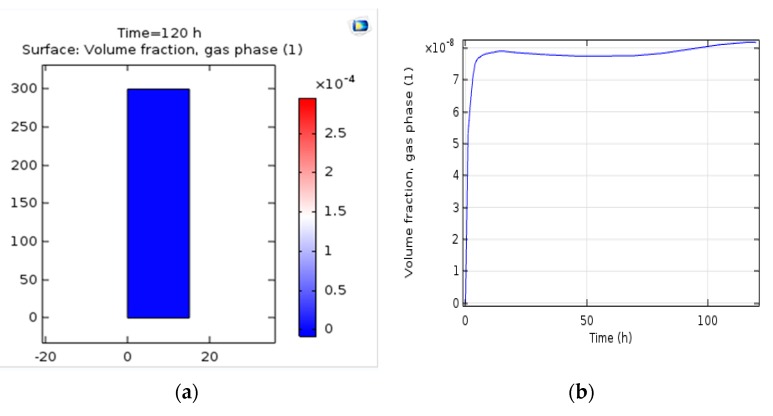

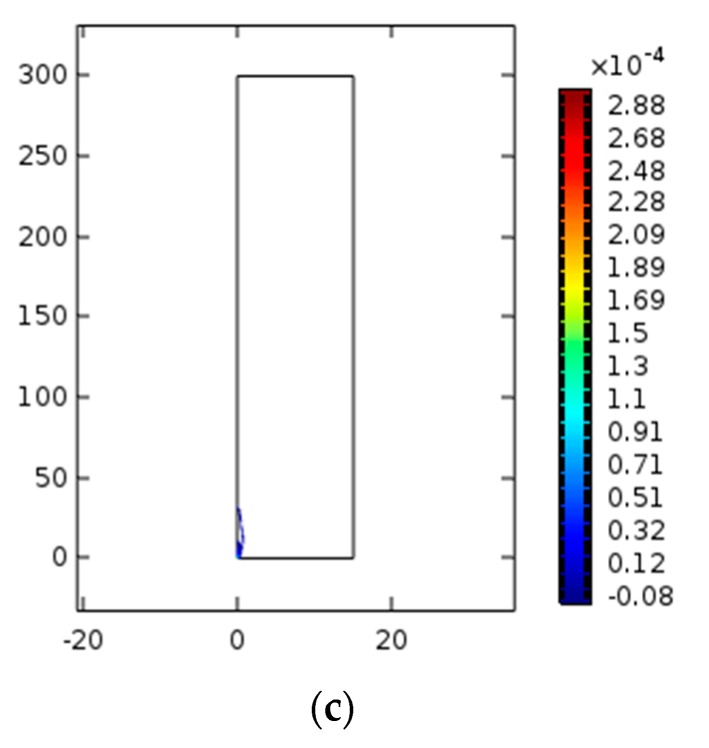
(**a**) The surface rate of gas accumulation along the reactor. (**b**) The rate of gas accumulation along the reactor. (**c**) Gas volume coefficient versus time.

Gas accumulation is a dimensionless key dimensional parameter for design purposes identifying the phenomenon of transition in bubble column systems (Equation (11)). It is basically defined as the volume fraction of gas-phase occupied by gas bubbles. Similarly, liquid and solid phases can be determined as a liquid and solid phase coefficient. All studies examined gas accumulation because it plays an essential role in the design and analysis of bubble columns. The volume of gas accumulation with the mathematical relation was studied. In similar, the results achieved in the simulation show the very low gas accumulation in the lower part of the reactor and around the central axis, which can be observed in Figure 11a,b. Along the reaction, gas accumulation increases throughout the reactor but it is false since the accumulated gas is eliminated from the upper end of the reactor and ultimately the amount of accumulated gas at the bottom of the reactor is computed. The graph of gas volume coefficient throughout the reactor can be seen in Figure 11c, such as the value of gas volume coefficient and their increase and decrease tendency with time and position.

ε_g=u_g/(0.3+2u_g)(11)

### 3.8. Shear Stress

**Figure 12:**
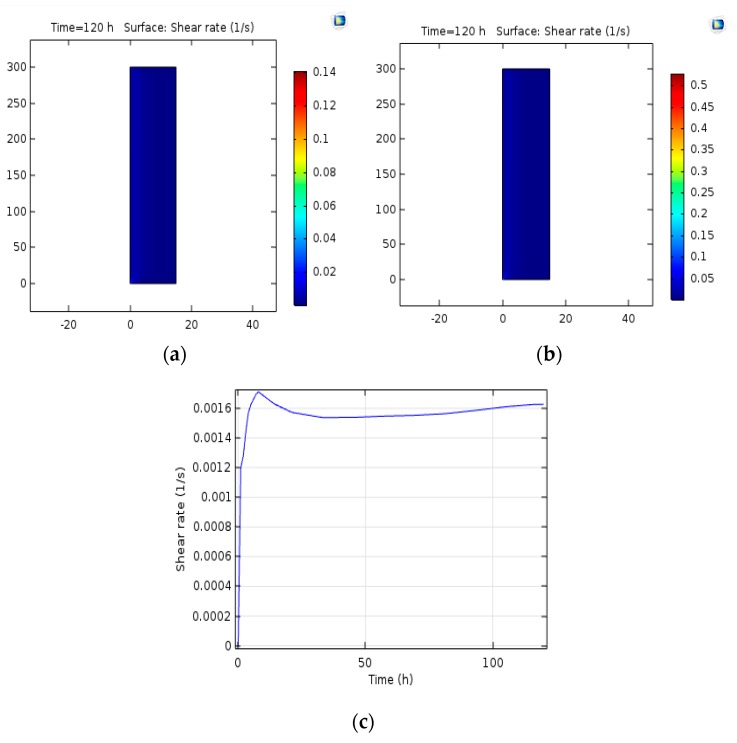
(**a**) The stress rate at the gas input velocity of 0.015 m/s. (**b**) The stress rate at the gas input velocity of 0.055 m/s. (**c**) The stress rate over time.

In the present project, the shear stress in different input flows was studied indicating (20) and (21) the stress rate, which is based on the scale available on the right of these shapes, the results achieved in line with the expectation of the sign, which contributes to an increase in the stress rate at the vicinity of the oven in line with the increase in the rate of intake gas and discharge. Figure 12 indicates the stress rate over time.

## 4. Discussion

### 4.1. The Results of Resolution Independence Analysis of Numerical Grid

As Table 2 shows, the variance in the three large, medium, and fine grid sizes is less than 1% indicating the independence of the obtained responses on the type and size of the grid. 

### 4.2. Concentration of Biomass

#### 4.2.1. Concentration Contour

Figure 3 shows that from the center toward the wall, concentrations are reducing being observed in Figure 3b. Thus, the largest value of production is located near the central axis. By enhancing the velocity of the areas around the center of the bioreactor, the maximum concentration, and with a distance less than the center of the percentage of production means that the increase in velocity would lead to a shorter spatial dimension to the final production.

Mousavi et al. examined the simulation of PHB production in an aircraft bioreactor with Fluent software. The average molecular concentration of PHB in the bioreactor was almost 0.331 and 0.3337 mol/L [36]. 

#### 4.2.2. Concentration Variations versus Time

Figure 5 shows the changes in the concentration of biomass were investigated vs. time. The final concentration of biomass is 14 mol/m^3^. The researchers showed the production of PHB was 8 g/L in a bubble column bioreactor at optimized conditions [17]. In addition, Shah Hosseini et al. applied a dynamic optimization program written in MATLAB software to specify the optimal amount of carbon and nitrogen sources on PHB production. The proposed model presented was confirmed on the experimental data. Using the preferred feed strategy, PHB was enhanced by 100% [37]. 

### 4.3. Velocity Contour 

Figure 7 shows the results of spatial changes in fluid velocity in the bioreactor. Dean et al. (2013) evaluated the use of large-spin simulations (LES) in mathematical simulations of microwave in bubble column reactors. The Euler–Euler approach is used to clarify two-phase motion equations that correspond slightly to the experimental data for both mid-velocity and motor velocities. LES provides better matching with simulation data through the k-ε model [38].

Mousavi et al. studied the fluid velocity fluctuations of the aircraft bioreactor in which the maximum fluid velocity can be seen in the input aperture at the bottom of the reactor. Similarly, the velocity of the fluid in the Down Comer is much lower than the Riser section [36]. 

Liquid velocity can directly effect production and methane gas enhances the growth structure of the biomass because these two parameters change in parallel. The flow rate can also change the behavior of the bioreactor and can change the production speed according to the size of the meshes designed [39,40].

### 4.4. Analysis of Variations in the Input Gas Velocity

The result of change of the gas flow rate inside the reactor vs. the number of bubbles and mass transfer rate (at four different velocities of 0.055, 0.015, 0.065, and 0.15 m/s) are displayed in Figure 8. As can be observed, as the gas flow rate increases, the content of biomass increases because of the increase in the number of bubbles and the number of encounters, which finally leads to an increase in the mass transfer rate and biomass production. The effects of gas and liquid velocity in a bubble pillar biomass with COMSOL software varying in the gas-liquid bubble column system, the velocity of gas and liquid with time and space in the column. Weather velocity vectors were achieved after a semi-stable mode at an input velocity of 0.001 m/s [41]. Such vectors indicated the velocity of the path along the path, which is useful in specifying the patterns of flow in the bubble column system. That contour indicated a gas concentration of 0.001 m/s. 

### 4.5. Effect of Changing the Bubble Diameter on the Concentration

Figure 9 shows that the concentration of the biomass is reduced by increasing the diameter of the bubble to reduce the flow rate. In addition, it enhances the final concentration of biomass by decreasing the diameter of the bubble. 

### 4.6. Pressure Analysis 

As it can be observed in Figure 10, the pressure across the reactor from bottom to top reduces because of the presence of gravity on the bubbles. 

### 4.7. Gas Accumulation

The graph of changes in gas volume throughout the reactor can be seen in Figure 11. In 2010, Mousavi et al. studied gas cumulating in aircraft bioreactors at numerous aeration rates [36]. The results obtained from the simulation were compared to the results of different mathematical relations related to the bubble columns and aircraft reactors. The simulation results are relatively suitable in comparison to the relationships and it approves the reliability of the simulation. For the aeration rate of 30 L/g. min, the mean diameter of the bubble was 5.1 mm [36].

Gas accumulation occurs during the five different phases of aeration. As expected, the amount of gas accumulation increased with aeration. The highest volume is always increased initially and then gas accumulation decreases because of the gas outflow from the bioreactor. However, in cases with higher aeration rates (40 and 50 L/min), the final amount of gas accumulation is higher than the peak value. In higher aeration conditions (40 and 50 L/min), the amount of gas accumulation is higher than the initial rate of gas accumulation in comparison to the lower aeration rate due to the accumulation of gas in the liquid phase.

### 4.8. Shear Stress

Wall shear stress is a critical parameter for determining energy transfer and movement in a two-phase flow system. The shear stress properties play an essential role in understanding the internal state of the two-phase flow because the liquid velocity and the highest grade of turbulence fluctuations are specified with the highest gradient. In order to clarify the mechanism of the two-phase bubble flow, it is significant to know the performance of the bubbles in the fluid flow. The movement of isolated bubbles seems to be related to the bubble distortion, the location of the injection point, and the average fluid flow rate. They found the only bubbles with diameters less than 5 mm along the wall while all the spherical bubbles and elliptical bubbles with a diameter greater than 5 milligram meters in the core of the stream [42,43]. Mousavi et al. in a study on shear stress in aircraft bioreactors specified the average shear stress in the bioreactor by applying the equations in FLUENT software for five different conditions. As expected, aeration enhances the shear stress in the bioreactor. Since airborne bioreactors and bubble columns fail at having a propellant, their tensions are lower than those of cohesive bioreactors, and the shear stress in the bioreactor is not likely to be significant, while the ability of the software to calculate shear stress is critical. The greatest shear stress relates to areas where gas is flowing from the opening. Vortices are located at the vicinity of upstream and downstream sections as well as the walls with higher shear stresses compared to other points [36]. 

## 5. Conclusions

In this study, a simulation of microorganisms capability for the production of PHB from natural gas (source of carbon) was carried out. The error interval is normally up to 20% for the difference between laboratory data and simulation because the resulting error can be because of simple simulation assumptions; thus, the simulation results were in good accordance with empirical results. Regarding the concentration contour, concentration is the lowest in the center of maximum concentration near the wall. The concentration gradient from the center is toward the wall and produces the highest percentage of production near the central axis of the bioreactor. Substrates and microorganisms with the environment were considered homogeneous, thus we would not have much difference in production compared to the place. Enhancing the gas flow rate has a direct relation to the production rate. Based on the three-velocity concentration-time graph, enhancing the intake rate of the agitator stirring increases and the percent of production enhances. The flow pattern in a bioreactor is dependent on the diameter of the bioreactor and the flow rate of the inlet gas. In this study, the bubble flow studied it placed due to the low velocity of the inlet gas (0.015 m/s) and the low diameter of the bioreactor (1.5 cm). Because of the concentration contour with enhancing the flux, the number of bubbles increases and results in more mass transfer and an increase in the value of biomass production. With increasing fluid velocity, a decrease can be seen in the amount of gas accumulation because liquid bubbles are rapidly displaced by liquid at higher velocities. Moreover, increasing fluid velocity decreases the duration of bubble stays.

Furthermore, based on the results in volume concentration, 50/50 from air and methane, the highest rate of microorganism growth and PHB production was achieved. According to presented graphs, such as time concentration and location concentration, the final amount of biomass production extracted was 1.6338 g/L under optimal conditions. The reason for selecting the volume of 50/50 was the coordination with the Taguchi algorithm that is based on research in the same conditions is the ideal conditions for evaluation of chosen sizes of the system. In addition, the amount of mesh size in the system was not significantly affected by the final concentration of production, so the ideal condition was regularly 1.6338 g/L. Thus, in different conditions of meshes, less than 1% difference was observed. In addition, the amount of gas accumulation decreases with increasing gas velocity.

## Figures and Tables

**Table 1:** Constants and values of the parameters inputted.

Constants	Value
Temperature	32 °C
Physical properties of the solute	discard
Velocity	Equal in all part
Situation of flow	Single-phase
Columnar bubble reactor	30 cm and 1.5 cm

**Table 2:** The number of elements in different groups to check the resolution independence of the numerical grid.

Mesh Size	Fine	Medium	Coarse
Number of elements	70,563	34,269	16,396

**Table 3:** Final concentration of biomass in the last step of solving equations in three computational grids.

Mesh Size	Fine	Medium	Coarse
Concentration (g/L)	1.63474	1.63338	1.63233

## Appendix A

List of symbols in equations:

**Parameters**	**Description**
$C_{\mathrm{PHB}}$	Dry weight of cell (g/L)
*K_d_*	Cell death rate (s/1)
μ	Growth rates for cell mass (1/s)
μ*_max_*	The maximum specific growth rate of microorganisms (1/s)
S	The concentration of limited substrate for growth (g/L)
Ks	Monod constant (g/L)
α	Fixed cell death time (s^−1^)
*K_d_* (∞)	Infinite cell death rate (s^−1^)
$u_{l}$	Velocity value in (m/s)
p	Pressure in (Pa)
*ϕ*	Volume fraction indicated with m^3^/m^3^
ρ	Density value with kg/m^3^
g	Gravity unit with m/s^2^
F	N/m^3^
μL	Dynamic velocity Pa.s
m_gl_	The mass transfer rate from gas to liquid (kg/m^3^)
U_slip_	Relative velocity
U_drift_	Drift velocity
M	The molecular weight of the gas (kg/mol)
R	Ideal gas constant (J/(mol·K) 3/3141472)
T	Temperature (K)
P_ref_	Scalar variable at (1 at or 101.325 Pa)
μ	Effective viscosity
